# Evaluation of effective coverage for type 2 diabetes in Mexican primary care health information systems: a retrospective registry analysis

**DOI:** 10.1186/s12939-023-01878-7

**Published:** 2023-04-05

**Authors:** Héctor Gallardo-Rincón, María Jesús Ríos-Blancas, Alejandra Montoya, Rodrigo Saucedo-Martínez, Linda Morales-Juárez, Ricardo Mujica, Alejandra Cantoral, Lorena Suarez Idueta, Rafael Lozano, Roberto Tapia-Conyer

**Affiliations:** 1 Carlos Slim Foundation, Lago Zurich 245, Presa Falcon Floor 20, Col. Ampliacion Granada, Miguel Hidalgo, 11529 Mexico City, Mexico; 2 National Institute of Public Health, Av. Universidad 655, Cuernavaca, 62100 México; 3 Health Department, Universidad Iberoamericana, Prolongación Paseo de Reforma 880, Lomas de Santa Fe, 01219 Mexico City, Mexico; 4 Institute for Health Metrics and Evaluation, University of Washington, 3980 15th Ave NE, Seattle, WA 98195 USA; 5 School of Medicine, National Autonomous University of Mexico, Av. Universidad 3000, Circuito Escolar CU, Edificio B 1Er Piso, Coyoacan, 04510 Mexico City, Mexico

**Keywords:** Effective coverage, Health care use, Mexico, Noncommunicable disease, Public health, Type 2 diabetes

## Abstract

**Background:**

As the leading cause of disability and the fourth leading cause of premature death in Mexico, type 2 diabetes (T2D) represents a serious public health concern. The incidence of diabetes has increased dramatically in recent years, and data from the Mexican National Health and Nutrition Survey (ENSANUT) indicate that many people remain undiagnosed. Persistent socioeconomic health care barriers exacerbate this situation, as T2D morbidity and mortality are worsened in vulnerable populations, such as those without social security. We evaluated the performance of public primary health centers (PHCs) in T2D medical attention through the measure of effective coverage (EC, a combined measure of health care need, use, and quality) at national, state, health jurisdiction, and municipality levels.

**Methods:**

This retrospective analysis used blinded data recorded during 2017 in the Non-communicable Diseases National Information System (SIC) and T2D prevalence reported in 2018 ENSANUT to evaluate the EC achieved. We included individuals ≥ 20 years old without social security who did not declare the use of private health care services. Each EC component (need, use, and quality) was estimated based on the Shengelia adapted framework. The Kruskal–Wallis test was applied to evaluate the associations among EC quintiles and demographics.

**Results:**

In 2017, 26.5 million individuals, aged ≥ 20 years, without social security, and without the use of private health care services, were under the care of 12,086 PHCs. The national prevalence of T2D was 10.3%, equivalent to 2.6 million people living with T2D in need of primary health care. Large contrasts were seen among EC components between and within Mexican states. We found that only 37.1% of the above individuals received health services at PHCs and of them, 25.8% improved their metabolic condition. The national EC was 9.3%, and the range (by health jurisdiction) was 0.2%–38.6%, representing a large geographic disparity in EC. We found an evident disconnect among need, utilization, and quality rates across the country.

**Conclusions:**

Expansion and improvement of EC are urgently needed to address the growing number of people living with T2D in Mexico, particularly in states with vulnerable populations.

**Supplementary Information:**

The online version contains supplementary material available at 10.1186/s12939-023-01878-7.

## Background

Type 2 diabetes (T2D) is the leading cause of disability and the fourth-leading cause of premature death in Mexico [1]. It is also one of the 10 most frequent reasons for hospitalization, and one of the main comorbidities affecting the health of the country’s population [2]. From 1990 to 2019, the number of people living with T2D in Mexico increased by 215%, from 3.8 million (95% confidence interval [CI] 3.5–4.1 million) to 11.9 million individuals (95% CI 10.9–12.8 million) [3]. This represents an increase in age-standardized prevalence from 8.2% (95% CI 7.6%–8.9%) to 10.3% (95% CI 9.5%–11.1%). The National Health and Nutrition Survey (ENSANUT, by its acronym in Spanish) estimated the prevalence of T2D by adding (1) people who already had a T2D diagnosis [4] and (2) undiagnosed people with fasting blood glucose levels > 126 mg/dL or glycated hemoglobin (HbA1c) > 6.5% measured at the moment of the survey [5]. Accordingly, almost 30% of the total prevalence represented undiagnosed cases. The same survey indicated that more than 50% of individuals who were aware of their condition remained at high risk for severe illness and complications because they never achieved control of their blood glucose levels, even though they received medical attention [4].

Across the country, the burden of disability and mortality owing to T2D remains higher for the most vulnerable populations [6, 7], reflecting persistent social and economic inequalities in the diagnosis, access to treatment, and control of this disease [8]. For instance, only 15.2% of adults with T2D reported that they had their HbA1c measured during the last year. Furthermore, there is a higher prevalence of uncontrolled T2D in the population without social security than in the population with social security [5]. People with diabetes who also face social inequalities are more likely to experience severe symptoms and complications of the disease, as well as comorbidities such as hypertension and obesity, than patients with higher socioeconomic status [9].

Providing equitable diabetes care in Mexico is challenging due to the fragmented health care system, which has diverse payers and providers [10]. The public sector includes social security institutions that provide services to formal sector workers, with both employers and employees contributing to health care funding, as is the case in Brazil, Chile, Spain, and Italy [11, 12]. For those without social security, the Mexican Ministry of Health is responsible for providing health care services and coverage on a public assistance basis funded by taxes. The private sector is financed through user payments and private health insurance premiums and offers services in private clinics and hospitals. This fragmentation makes it difficult to coordinate care and identify gaps in diabetes care coverage, which can result in inequitable health outcomes for patients [10].

In this study, we aimed to evaluate the performance of public primary health centers (PHCs) administered by the Mexican MOH, estimating the T2D effective coverage (EC) and its components—need, use, and quality—among people 20 years of age and older in Mexico with no social security who did not report the use of private health care services. Additionally, we aimed to evaluate the possible heterogeneity among geographic regions.

## Methods

### Study design

We conducted a retrospective analysis to estimate the EC achieved in 12,086 PHCs that provided health care to people without social security who were nonusers of private health services during 2017 within the 32 states, 245 health jurisdictions, and 2,457 municipalities of Mexico.

### Information sources

We identified the population without social security who were nonusers of private health services based on the National Intercensal Population Survey 2015 and aggregated it at national, state, health jurisdiction, and municipality levels [13]. We considered a conservative scenario, assuming that the proportion of the population without social security and nonusers of private health services has minimal change between 2015 and 2017.

The T2D prevalence, reported by ENSANUT 2018 [14], was used to estimate the number of people living with T2D at different geographic levels, considering the survey sampling design. The National Health Survey System in Mexico includes a series of multi-thematic surveys on health and nutrition [15], which has been conducted in 2006 [16], 2012 [17], 2016 [18], and 2018 [19]. ENSANUT 2018 is probabilistic, stratified, and clustered at the household level. The sampling method is described in detail elsewhere [19]. Briefly, it included 1,580 households per state and 50,654 at the national level; thus, the information is representative at the national level and for all 32 states. The health jurisdiction and municipality geographical division of the country in the year 2017 was considered, which includes 245 jurisdictions and 2,457 municipalities [20].

Information about the use, quality, and outcomes of medical care for patients with T2D was obtained from the national nominal system of chronic disease (SIC, for the acronym in Spanish)[21], which gathered data from more than 12,000 PHCs of the MOH in 2017. The SIC is a census used to register data of medical care given to patients, including activities implemented under the normative regulations, prescribed treatments, and biomarker measurements for monitoring control. The SIC also includes sociodemographic information such as sex, age, and clinical and family history for each patient who attends any MOH PHC nationwide. The use of data was approved by the Mexican MOH, and the databases were blinded before access and analyzed at the aggregate level, so informed consent was not required.

Information about the demographics and poverty determinants was obtained from the poverty measurement indicators of National Council for the Evaluation of Social Development Policy (CONEVAL, for the acronym in Spanish) [22].

### Statistical analysis

The measurement of EC was assessed based on the original proposal by Shengelia et al. framework [23] using the components of need, utilization, and quality of health care interventions, as follows:

$$\mathrm{EC}=\mathrm{Q}*\mathrm{U}|{\mathrm{N}=1}$$

In the original framework, EC is the effective coverage; *N* = 1 is the true need for receiving health care services; U is the utilization of health care services and refers to the probability that the individual with a need will receive the intervention; and Q is the quality or health gain ratio of the gain provided to the person by an intervention in relation to the maximum possible health gain [23].

In this study, we estimated the need (N) by multiplying T2D prevalence by the population under the responsibility of each PHC (individuals without social security who were nonusers of private health care services). The utilization (U) was estimated as the proportion of individuals with the need who sought T2D medical attention (visits or follow-up treatment) at any PHC.

Quality was measured based on the clinical goal of metabolic improvement (health gain) in HbA1c [24, 25], as the proportion of individuals who achieved metabolic control (baseline HbA1c ≥ 7%, follow-up HbA1c < 7%) or maintained control (baseline HbA1c < 7%, follow-up HbA1c < 7%) from baseline. Baseline was defined as the last HbA1c measurement within 6 months before the first consultation in 2017 and was compared with a follow-up measurement recorded within 6 months of the patient’s final consultation. We opted for a conservative scenario, assuming that those with insufficient information to evaluate their metabolic control did not achieve control.

Each EC component was estimated by national, state, health jurisdiction, and municipal levels. We present means and ranges nationally, by state, and by health jurisdiction. We also analyzed the differences in EC according to geographic distribution, mapping at state, health jurisdiction, and municipality levels. Finally, municipalities were grouped in quintiles according to the EC distribution of the estimations and we evaluated the correlation of each quintiles of EC with demographics (population size, population density) and poverty determinants (population with low education, population without access to health services, and population lacking basic sanitation services) by the Kruskal–Wallis test. All statistical analyses were performed with Stata 15 statistical software (StataCorp LLC, College Station, TX, USA).

## Results

The estimated population aged ≥ 20 years in Mexico with no social security who reported no use of private health care services was 26.5 million in 2017. This population was considered to be under the primary medical care responsibility of 12,086 MOH PHCs in the country. The national prevalence of T2D was 10.3% (95% CI: 9.9%–10.7%) according to the ENSANUT 2018 (reported prevalence within the population with no social security), which is equivalent to 2.6 million people living with T2D and who potentially needed primary health care (Table 1).

**Table 1:** Description of the study population under the responsibility of PHCs and effective coverage components at national and state levels

					**Effective coverage dimensions**			
					**Need** **(*N* = 1)**	**Utilization** **(U)**	**Quality** **(Q)**	**Effective coverage** **(EC)**
	**A**	**B**	**C**	**D**	**E**	**F**	**G**	**H**
	**Health jurisdictions**	**MOH PHCs**	**Population 20 + without social security**	**Prevalence (%)**	Population 20 + who needs medical care for T2D	Percentage of population who needed and received medical care for T2D	Percentage of population 20 + who received medical care and improved metabolic condition	EC = Q × U | *N* = 1
					E = D*C			
	N	N	N	Mean	Mean	Mean	Mean	Mean
			(HJ minimum-HJ maximum)	(HJ minimum-HJ maximum)	(HJ minimum-HJ maximum)	(HJ minimum-HJ maximum)	(HJ minimum-HJ maximum)	(HJ minimum-HJ maximum)
**National**	**245**	**12,086**	**26,534,076** **(5–322,557)**	**10.3** **(6.0–16.1)**	**16,717** **(595–47,073)**	**37.9** **(5.3–94.2)**	**25.8** **(1.5–82.1)**	**9.5** **(0.2–38.6)**
Aguascalientes	3	91	218,184 (3,641–103,644)	7.6 (7.3–9.2)	7,600 (2,117–9,954)	53.5 (47.6–63.6)	20.9 (16.9–23.1)	11.1 (8.0–11.5)
Baja California	3	174	449,493 (18,465–182,327)	10.6 (9.8–11.5)	17,927 (9,577–23,397)	40.7 (36.5–42.4)	23.1 (9.6–49.0)	9.0 (4.1–17.9)
Baja California Sur	4	60	117,218 (4,542–41,560)	9.8 (7.5–14.8)	4,069 (1,147–25,857)	37.7 (10.0–42.6)	36.6 (32.8–59.0)	13.7 (3.3–19.3)
Campeche	3	140	256,050 (4,678–53,819)	13.8 (12.9–14.3)	14,245 (7,798–19,549)	33.8 (29.3–43.8)	16.8 (13.9–24.6)	5.7 (4.1–8.4)
Chiapas	10	740	1,702,440 (452–113,127)	8.1 (6.0–9.8)	17,120 (4,051–28,079)	20.1 (10.0–43.9)	12.7 (1.5–32.8)	2.7 (0.2–6.9)
Chihuahua	10	255	508,213 (346–118,070)	9.9 (8.3–12.9)	10,658 (1,034–25,857)	53.5 (10.0–75.3)	27.1 (7.8–39.7)	14.3 (3.3–29.4)
Mexico City	16	223	1,348,687 (24,691–322,557)	12.2 (9.1–14.1)	18,425 (2,247–41,287)	32.4 (24.5–74.0)	40.3 (29.3–50.7)	12.8 (9.0–30.2)
Coahuila de Zaragoza	8	148	258,925 (256–45,800)	12.5 (9.8–14.1)	6,059 (595–25,857)	10.5 (6.5–46.6)	45.9 (21.7–76.6)	4.9 (1.5–14.0)
Colima	3	127	162,196 (2,834–40,542)	11.3 (10.9–11.6)	6,726 (3,803–8,540)	39.5 (24.0–47.0)	15.7 (8.6–18.3)	6.5 (2.1–8.6)
Durango	4	167	367,341 (650–108,493)	10.9 (9.8–13.1)	13,957 (3,265–25,857)	25.5 (10.0–46.3)	14.5 (4.8–32.8)	3.2 (2.2–4.4)
Guanajuato	8	443	1,565,296 (2,814–245,217)	9.8 (8.6–11.9)	20,070 (14,576–24,864)	57.5 (5.3–81.3)	44.8 (39.9–51.3)	25.8 (2.7–36.2)
Guerrero	7	951	1,293,050 (2,558–190,446)	11.2 (8.9–12.9)	25,393 (13,826–47,073)	46.4 (34.3–55.8)	14.0 (10.5–15.8)	6.4 (3.9–8.2)
Hidalgo	17	550	886,803 (1,349–50,632)	12.9 (11.9–14.3)	8,226 (1,757–13,080)	29.4 (15.3–39.5)	24.8 (15.8–35.1)	7.3 (3.4–11.9)
Jalisco	13	740	1,457,426 (979–189,524)	7.8 (6.5–9.5)	10,054 (2,545–16,797)	46.6 (15.3–93.2)	38.0 (19.4–58.3)	15.8 5.3–38.6)
Michoacán de Ocampo	8	482	1,109,399 (1,343–116,072)	10.0 (9.3–11.2)	15,833 (5,315–25,857)	38.9 (10.0–48.4)	9.0 (3.2–32.8)	3.4 (0.9–5.2)
Morelos	3	206	538,891 (3,337–73,053)	12.1 (12.0–12.5)	24,719 (10,763–28,714)	26.8 (16.3–36.0)	16.1 (10.0–17.6)	4.4 (1.6–6.1)
Mexico State	19	1,078	3,409,474 (1,464–245,434)	9.4 (8.3–11.0)	20,212 (9,163–30,035)	37.4 (10.0–73.0)	17.4 (6.0–33.4)	6.2 (1.5–16.5)
Nayarit	3	208	285,409 (3,183–54,044)	10.7 (9.7–12.2)	15,386 (8,531–26,748)	22.5 (21.2–23.2)	46.9 (42.4–53.4)	10.5 (9.8–11.3)
Nuevo León	8	431	513,227 (379–106,915)	13.2 (10.4–16.1)	10,526 (3,325–17,213)	28.3 (18.6–45.6)	43.8 (34.9–61.7)	12.3 (6.5–18.5)
Oaxaca	6	802	1,307,705 (5–53,066)	10.2 (9.2–11.7)	24,093 (10,115–36,140)	35.5 (10.0–70.5)	6.1 (1.7–32.8)	1.6 (0.6–3.3)
Puebla	10	673	1,625,491 (172–238,031)	9.1 (7.8–10.2)	17,355 (4,282–25,857)	36.0 (10.0–56.7)	43.9 (27.2–82.1)	14.9 (3.3–26.3)
Querétaro	4	252	407,080 (4,444–107,661)	7.7 (7.1–8.2)	9,450 (3,393–12,045)	71.9 (59.8–94.2)	18.3 (12.7–35.0)	13.1 (9.2–28.3)
Quintana Roo	3	185	277,187 (6,806–76,725)	8.6 (7.8–9.9)	9,210 (5,166–11,819)	79.2 (71.0–81.1)	18.3 (11.6–31.2)	15.6 (9.4–23.5)
San Luis Potosí	7	291	714,735 (722–113,474)	10.9 (9.8–12.7)	12,078 (6,835–25,857)	45.5 (10.0–51.0)	28.0 (20.3–32.8)	12.6 (3.3–14.0)
Sinaloa	6	266	531,099 (6,930–93,946)	10.8 (10.2–11.9)	10,871 (4,288–14,975)	27.6 (19.2–33.7)	26.0 (12.8–41.7)	6.8 (3.7–9.5)
Sonora	6	207	420,723 (152–88,660)	12.2 (9.8–13.2)	10,386 (2,603–25,857)	25.6 (10.0–38.1)	24.7 (14.1–32.8)	6.6 (2.5–11.2)
Tabasco	17	584	766,993 (13,685–143,183)	11.5 (9.2–13.0)	13,514 (1,779–47,073)	47.1 (31.2–76.2)	26.3 (8.9–79.0)	11.7 (5.0–26.1)
Tamaulipas	12	296	641,955 (633–82,712)	14.0 (12.8–16.0)	8,990 (2,841–13,363)	34.8 (26.4–47.3)	32.3 (13.5–76.8)	10.7 (4.1–23.2)
Tlaxcala	3	123	433,665 (1,235–33,366)	9.2 (8.7–9.5)	15,412 (7,600–20,838)	62.5 (57.9–70.3)	17.7 (17.0–19.4)	9.8 (8.3–12.1)
Veracruz	11	793	2,038,825 (597–69,718)	11.6 (9.8–12.8)	23,174 (15,434–29,281)	26.0 (10.0–30.9)	34.6 (24.2–67.9)	8.8 (3.3–14.2)
Yucatán	3	166	451,156 (149–92,967)	10.4 (9.8–11.0)	16,984 (7,845–25,857)	39.8 (10.0–49.4)	23.8 (18.6–32.8)	9.0 (3.3–9.9)
Zacatecas	7	234	469,740 (245–58,991)	10.9 (9.6–12.5)	8,775 (3,070–25,857)	40.0 (10.0–55.1)	14.7 (6.3–32.8)	5.6 (2.6–9.4)

Ranges in parentheses are those at the HJ level

*HJ* Health jurisdiction, *MOH* Ministry of Health, *PHC* Primary health center, *T2D* Type 2 diabetes

**Fig. 1:**
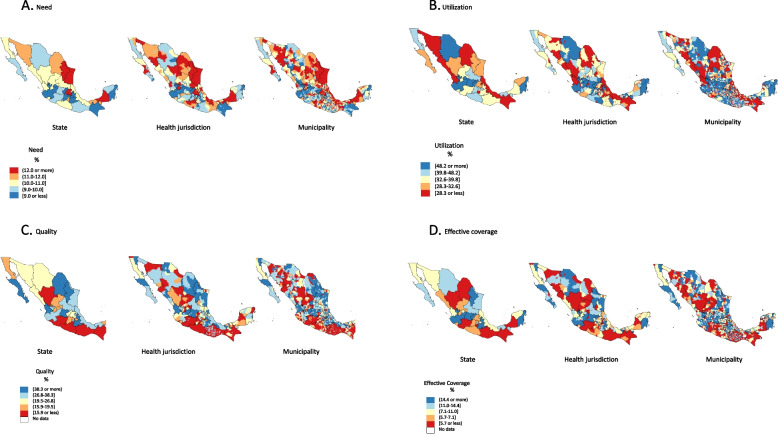
Components of effective coverage for type 2 diabetes in the Mexican population without social security. Data are color-coded by quintiles and shown according to state, health jurisdiction, and municipality. Source: Mexican Ministry of Health primary health centers, 2017. **a** Need: prevalence of type 2 diabetes multiplied by the estimated population under the responsibility of a primary health center; **b** Utilization: proportion of estimated population under the responsibility of a primary health center that sought care for type 2 diabetes; **c** Quality: proportion of patients who achieved metabolic improvement among those who sought care; and **d** Effective coverage: EC = Q × U | *N*=1, where *N* = 1 is the true need for receiving health care services; U is the utilization of health care services and refers to the probability that the individual with a need will receive the intervention; and Q is the quality, or health gain ratio of the gain provided to the person by an intervention in relation to the maximum possible health gain

Among states, the average percentage of the population requiring medical care for T2D ranged from a minimum of 7.6% (within-state range: 7.3%–9.2%) in Aguascalientes to a maximum of 14.0% in Tamaulipas (within-state range: 12.8%–16.0%). Notably, some northern states, such as Nuevo León, Tamaulipas, Sonora, and Coahuila, and states located around the Gulf of Mexico, such as Veracruz, Tabasco, and Campeche, presented the highest need in the country (Fig. 1A). Among health jurisdictions, the need ranged from 6.0% in Ocosingo, Chiapas to 16.1% in Monterrey, Nuevo Leon (Supplementary Table 1, Fig. 1A).

According to the information recorded in the SIC during 2017, 998,135 individuals (283,706 men and 714,429 women) sought at least one medical consultation in their respective MOH PHCs, meaning that only 37.1% of the base population with T2D who needed health services at the PHCs received medical attention. Across the country, states with the highest proportion of the population that received health care for T2D were Quintana Roo (79.2%), Querétaro (71.9%), Guanajuato (53.7%), and Chihuahua (53.5%). We observed the lowest utilization in the states of Coahuila (10.5%), Chiapas (20.1%), Nayarit (22.5%), and Durango (25.5%) (Table 1, column F). The within-state variability in utilization among health care jurisdictions ranged between 5.3% and 94.2% (Supplementary Table 1, Fig. 1B).

The change in metabolic glucose levels and T2D control (quality) was assessed for 584,899 adults (13.6% of patients diagnosed with T2D) because these individuals had at least two measurements (baseline and follow-up). Among the total population, 26.6% improved their metabolic condition. Importantly, 43% of the population who attended a PHC more than once still had an uncontrolled or worsened metabolic condition. The states with the best performance, on average, were Nayarit, Coahuila, Guanajuato, Puebla, Nuevo León, and Mexico City, all of which had values over 40% (Table 1, column G). In contrast, Oaxaca, Michoacán, Chiapas, Guerrero, Durango, and Zacatecas presented the worst capability concerning improving the health of people living with T2D (under 15%). This indicator showed the greatest variability among health jurisdictions, ranging from 1.5% to 82.1% (Supplementary Table 1) (Fig. 1C).

When we jointly analyzed the components of need, utilization, and quality, we estimated that the EC achieved in MOH PHCs at the national level was 9.5% (within-health jurisdiction range: 0.2%–38.6%). The states with the lowest EC were Chiapas, Durango, Michoacán, and Oaxaca; states with the highest EC were Guanajuato, Jalisco, Puebla, Quintana Roo, Puebla, and Chihuahua (Table 1, column H).

**Fig. 2:**
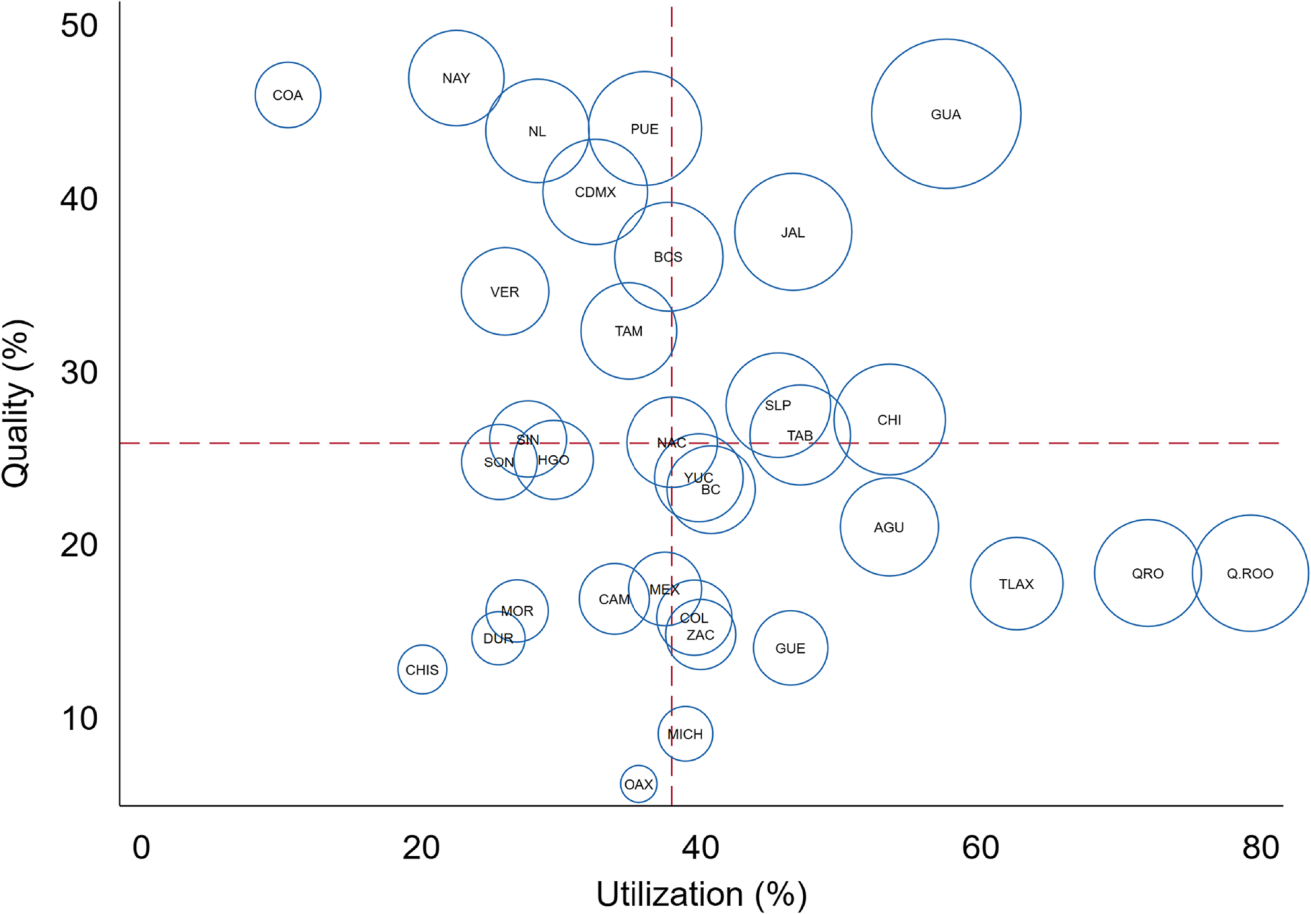
Utilization and quality of care for type 2 diabetes in Mexico, by state. The size of the circles represents the level of effective coverage achieved. Abbreviations: AGU, Aguascalientes; BC, Baja California; BCS, Baja California Sur; CAM, Campeche; CHIS, Chiapas; CHI, Chihuahua; CDMX, Ciudad de México; COA, Coahuila de Zaragoza; COL, Colima; DUR, Durango; GUA, Guanajuato; GUE, Guerrero; HGO, Hidalgo; JAL, Jalisco; MICH, Michoacán de Ocampo; MOR, Morelos; MEX, México; NAC, National level; NAY, Nayarit; NL, Nuevo León; OAX, Oaxaca; PUE, Puebla; QRO, Querétaro; QROO, Quintana Roo; SLP, San Luis Potosí; SIN, Sinaloa; SON, Sonora; TAB, Tabasco; TAM, Tamaulipas; TLA, Tlaxcala; VER, Veracruz de Ignacio de la Llave; YUC, Yucatán; ZAC, Zacatecas

We observed different performance patterns concerning the EC achieved among states, according to the relationship between PHC utilization and T2D control (quality). Figure 2 shows that those states with lower utilization and lower T2D control (Chiapas, Durango, Morelos, and Oaxaca) also had the lowest EC (lower left quadrant). States in the upper left quadrant (Mexico City, Nuevo León, Nayarit, Coahuila, and Veracruz) represent those states with lower utilization but better T2D control, with an EC close to the national value. States with higher utilization but poorer T2D control (Aguascalientes, Querétaro, Guerrero, Quintana Roo, and Tlaxcala) are shown in the lower right quadrant of Fig. 2. Finally, states with the best performance, i.e., with higher utilization and better T2D control (Jalisco and Guanajuato), are shown in the upper right quadrant of the figure.

Important differences in EC can be observed within regions of Mexico at the municipality level (Fig. 1D). The correlation analysis revealed that the highest quintiles of EC showed an inverse and statistically significant correlation with population size, population density, population with low education, population without access to health services, and population lacking basic sanitation services (Table 2).

**Table 2:** Correlation analysis of effective coverage and social health determinants by municipality

	**Quintiles of effective coverage**					
**Characteristic**	**Q1**	**Q2**	**Q3**	**Q4**	**Q5**	***p*-value (Kruskal–Wallis test)**
	**Mean (min – max)**	**Mean (min – max)**	**Mean (min – max)**	**Mean (min – max)**	**Mean (min – max)**	
Effective coverage (%)	1.6 (0.1–3.2)	4.9 (3.2–6.5)	8.7 (6.5–10.9)	14.4 (10.9–19.1)	34.4 (19.2–100.0)	
Population	45,425 (537–1,679,610)	68,576 (345–1,789,531)	84,479 (234–1,503,505)	67,799 (375–1,815,551)	23,029 (288–610,700)	0.0001
Population density	187.0 (0.8–7882.5)	371.3 (0.3–12,494.2)	521.6 (0.5–16,435.4)	398.3 (0.1–16,898.2)	126.9 (0.4–7856.0)	0.0001
Population with multidimensional poverty (%)	70.5 (19.0–97.3)	65.5 (21.4–97.4)	63.4 (12.8–97.0)	64.6 (8.7–96.6)	67.5 (14.3–96.4)	0.0001
Population with low education (%)	32.3 (8.2–62.5)	29.1 (8.7–53.1)	28.3 (5.4–59.5)	28.6 (3.7–61.1)	30.9 (4.8–65.1)	0.0001
Population without access to health services (%)	15.5 (1.9–50.2)	14.6 (2.0–40.8)	13.7 (3.0–39.3)	13.3 (2.0–34.6)	12.9 (0.9–77.4)	0.0001
Population lacking basic sanitation services (%)	50.1 (0.3–99.6)	43.1 (0.1–99.9)	41.6 (0.1–98.7)	39.0 (0.0–100.0)	43.0 (0.0–100.0)	0.0001

## Discussion

This report provides evidence of the three components of EC—need, use, and quality—in a population with no social security who are nonusers of private services, at national, state, health jurisdiction, and municipality levels. Our results suggested that there is an urgent need to expand and improve the EC of T2D as part of policies to reduce the burden of disease and health vulnerability in the Mexican population.

Several studies have demonstrated that EC is a good indicator to quantify the improvement of the health of the population who receive one or more interventions from the health care system when needed [26–30]. In the Latin America region, Mexico was the first country that measured the EC of the health care system at the national and state levels through 18 basic health programs; in the following years several studies to evaluate EC at the state level have been conducted in Mexico [30–35]. The present study reports the first evaluation of T2D in the population with no social security coverage and nonusers of private health care services at state, health jurisdiction, and municipality levels using the SIC, the first nominal registry that tracks patient health information. The results could be useful not only to understand the effectiveness of interventions, but also to provide practical information to improve PHC services [36].

For this analysis, we excluded individuals who reported seeking health care services through the private sector, which represents almost 50% of the population without social security [14, 37]. According to Colchero et al., between 2004 and 2018 in Mexico, the membership to health services for the nonsocial security population grew almost 10 times, from 4.8 to 42.0 million people, but this increase was not accompanied by an equivalent increase in the availability of public health care services [38]. In contrast, the availability of private health care services grew rapidly, mostly by offices adjacent to pharmacies. Such offices have been successfully competing with public options at the primary level of care in Mexico, which contributes to the low use of public services. However, there is no regulation for private facilities, and the quality and effectiveness of private care is not known [38].

We estimated that there were 2.6 million individuals without social security living with T2D in Mexico, who mainly depend on MOH PHCs for medical care. Most of this population is therefore expected to receive treatment and follow-up care according to the Mexican health system, which is obligated to guarantee universal health access to all Mexican citizens within their communities [39]. Nevertheless, we found an evident disconnect among need, utilization, and quality rates across the country.

The greatest need was found in the health jurisdictions and municipalities located mainly in northern Mexico and around the Gulf of Mexico. However, most of the jurisdictions in those regions achieved a low to moderate rate of utilization. In contrast, health jurisdictions in Nuevo León and Mexico City were classified among the top 10 states in which patients maintained, achieved, or improved T2D metabolic control, despite those states having the lowest rates of PHC utilization. Fortunately, some jurisdictions in states such as Jalisco achieved both high rates of utilization and high quality of care (health gain).

This analysis revealed that glycemic control was generally poor among individuals with T2D; this finding highlights that immediate action to improve the quality of primary health care is required. International evidence has shown that the lack of metabolic control increases the probability of complications, which can lead to economic losses owing to absence from work, hospitalization, and premature death [32]. Therefore, indicators related to improving health care must be monitored in primary care through preventive measures and timely diagnosis and treatment of patients [35, 40, 41]. Although it is well established that longer duration of T2D is associated with poor glycemic control and worse self-care [42, 43], disease duration was not included in the present analysis and thus somewhat limits our interpretation of the results. The concept of ambulatory care-sensitive hospitalization (ACSH) can also be applied to assess the impact of adequate T2D care on the economic factors listed above. ACSH (hospitalization that could be prevented by adequate intervention in primary care) for T2D complications has increased greatly in Mexico in recent years, and the financial costs and increased health burden related to ACSH suggest that improvements in primary care (and thus, EC) could considerably ease this burden [44, 45]. Nowadays, the second-highest cause of ACSH at the national level in Mexico is T2D and non-communicable diseases, representing more than 30% of total consultations in the age group above 50 years [46].

In this analysis, we found that some factors were correlated with EC at the municipality level, such as the lack of access to health services and lack of sanitation. These results are consistent with those previously reported in other studies [40, 41].

Even though our analysis did not assess early detection of T2D, we recognize that early detection presents one of the greatest challenges to overcome and is an area where the health care system must take an active role through timely screening. Previously we showed that screening strategies for pre-disease states (such as pre-T2D) are crucial in the continuum of care and ideally should be included as part of the effective coverage [47]. We identified in a large population size analysis that 13.4% of the screened population presented this condition.

One strength of this analysis is that the quality component of EC was assessed using the biomarker HbA1c, similar to previous studies that used HbA1c to assess T2D EC [48]. An additional strength for the other two components (utilization and quality) is that they were measured consistently and taken from the SIC registry designed for this purpose (ex-ante approach).

One limitation of this study is that T2D control could only be assessed for the 584,899 adults who had HbA1c data available. Furthermore, although we attempted to estimate prevalence by age and sex, these calculations were not sufficiently precise at the municipality level to draw meaningful conclusions. Another of the limitations is that we adopted a conservative scenario, which assumed minimal changes in the proportion of the population without social security and non-users of the private sector between 2015 and 2017. However, this assumption may have influenced our estimates, indicating the need for further research to examine the potential impact of changes in this population on the findings of future studies. Another weakness of the analyses is that we included routine secondary source data from ENSANUT to estimate need (ex-post approach).

The combination of ex-ante and ex-post sources of information allowed us to make estimations not only at the state level but also at the health jurisdictional and municipality levels. Health information systems are useful in providing routine data for administrative and clinical purposes and are key tools in assessment of EC [36]. It has been shown that electronic health records can be used to evaluate clinic performance and interventions in Mexico [49]. Furthermore, as shown in this analysis, the combination of health information systems with population data results is a useful tool for benchmarking the performance of PHCs at national, state, health jurisdictional, or municipality levels. Thus, the present results are beneficial for health authorities and decision-makers to prioritize and focus on developing appropriate local health policies.

## Conclusion

Our findings provide a baseline for identifying areas to improve access, use, and quality of care among the Mexican population without social security. Large differences between users of health care services at the health jurisdiction and municipality levels suggest that a next step would be to take actions to increase the use of health services. Given the complexity of factors related to control of T2D in Mexico, and the segmented health care system, efforts within the MOH must be accompanied by intersectoral action to strengthen prevention and to ensure access to high-quality care. The lessons learned from this study can be used to promote the use of EC as a routine indicator for monitoring the performance of health care systems across the country.

## Supplementary Information

**Additional file 1: Supplementary Table 1.** Effective coverage components at the health jurisdictional level.

## Data Availability

All data generated or analyzed during this study are included in this published article. Such data are publicly available upon request from the Mexican Health Minister.

## Abbreviations

ACSH: Ambulatory care-sensitive hospitalization
CI: Confidence interval
EC: Effective coverage
ENSANUT: National Health and Nutrition Survey
MOH: Ministry of Health
PHC: Public primary health center
SIC: Non-communicable Diseases Information System
T2D: Type 2 diabetes
WHO: World Health Organization
