# Supplementary material for: Evaluation of effective coverage for type 2 diabetes in Mexican primary care health information systems: a retrospective registry analysis
Source: Int J Equity Health. 2023 Apr 5;22:61. doi: 10.1186/s12939-023-01878-7 (PMC10074724; doi:10.1186/s12939-023-01878-7)
Supplement: Supplementary file 1 — Additional file 1: Supplementary Table 1. Effective coverage components at the health jurisdictional level. [file 12939_2023_1878_MOESM1_ESM.docx]

**Supplementary Table 1.** Effective coverage components at the health jurisdictional level.

|  |  |  |  | **Effective coverage dimensions** | | | |
| --- | --- | --- | --- | --- | --- | --- | --- |
|  |  |  |  | **Need (N = 1)** | **Utilization (U)** | **Quality (Q)** | **Effective coverage (EC)** |
|  |  |  |  |  |  |  |  |
|  | **B** | **C** | **D** | **E** | **F** | **G** | **H** |
| **Health jurisdiction** | **MOH PHCs** | **Population 20+ without social security** | **Prevalence** | Population 20+ who needs medical care for T2D | Percentage of population who needed and received medical care for T2D | Percentage of population 20+ who received medical care and improved metabolic condition | EC = Q × U \| N=1 |
|  |  |  |  | E=D*C |  |  |  |
|  | N | N | Mean | Mean | Mean | Mean | mean |
|  |  |  |  |  |  |  |  |
| **Aguascalientes** | **91** | **218,184** | **7.6** | **7,600** | **53.5** | **20.9** | **11.1** |
| Aguascalientes | 46 | 134,708 | 7.4 | 9,954 | 49.9 | 23.1 | 11.5 |
| Calvillo | 13 | 23,007 | 9.2 | 2,117 | 47.6 | 16.9 | 8.0 |
| Rincon De Romos | 32 | 60,469 | 7.3 | 4,442 | 63.6 | 17.6 | 11.2 |
|  |  |  |  |  |  |  |  |
| **Baja California** | **174** | **449,493** | **10.6** | **17,927** | **40.7** | **23.1** | **9.0** |
| Ensenada | 48 | 97,727 | 9.8 | 9,577 | 42.3 | 20.0 | 8.4 |
| Mexicali | 61 | 128,451 | 11.5 | 14,772 | 36.5 | 49.0 | 17.9 |
| Tijuana | 64 | 223,315 | 10.5 | 23,397 | 42.4 | 9.6 | 4.1 |
|  |  |  |  |  |  |  |  |
| **Baja California Sur** | **60** | **117,218** | **9.8** | **4,069** | **37.7** | **36.6** | **13.7** |
| Comondu | 12 | 19,236 | 14.8 | 2,847 | 38.8 | 37.1 | 14.4 |
| La Paz | 23 | 41,560 | 9.7 | 4,031 | 42.6 | 34.0 | 14.5 |
| Los Cabos | 17 | 40,740 | 7.5 | 3,056 | 36.5 | 33.2 | 12.1 |
| Mulege | 8 | 11,140 | 10.3 | 1,147 | 32.8 | 59.0 | 19.3 |
|  |  |  |  |  |  |  |  |
| **Campeche** | **140** | **256,050** | **13.8** | **14,245** | **33.8** | **16.8** | **5.7** |
| Campeche | 71 | 139,178 | 14.0 | 19,549 | 29.3 | 13.9 | 4.1 |
| Carmen | 26 | 56,612 | 14.3 | 8,068 | 34.1 | 24.6 | 8.4 |
| Escarcega | 43 | 60,260 | 12.9 | 7,798 | 43.8 | 16.1 | 7.0 |
|  |  |  |  |  |  |  |  |
| **Chiapas** | **740** | **1,702,440** | **8.1** | **17,120** | **20.1** | **12.7** | **2.7** |
| Comitan | 69 | 234,795 | 8.6 | 20,305 | 23.7 | 12.0 | 2.8 |
| Motozintla | 36 | 57,825 | 7.0 | 4,051 | 20.4 | 9.3 | 1.9 |
| Ocosingo | 89 | 109,085 | 6.0 | 6,557 | 12.6 | 1.5 | 0.2 |
| Palenque | 87 | 151,026 | 7.6 | 11,411 | 14.8 | 11.4 | 1.7 |
| Pichucalco | 77 | 141,964 | 7.8 | 11,121 | 21.5 | 24.3 | 5.2 |
| San Cristobal De Las Casas | 146 | 224,494 | 7.1 | 15,953 | 14.4 | 5.5 | 0.8 |
| Tapachula | 60 | 240,792 | 8.6 | 20,804 | 21.2 | 10.4 | 2.2 |
| Tonala | 47 | 75,199 | 9.7 | 7,288 | 43.9 | 15.7 | 6.9 |
| Tuxtla Gutierrez | 96 | 332,544 | 8.4 | 28,079 | 16.8 | 15.0 | 2.5 |
| Villaflores | 33 | 118,164 | 8.7 | 10,336 | 28.4 | 21.4 | 6.1 |
|  |  |  |  |  |  |  |  |
| **Chihuahua** | **255** | **508,213** | **9.9** | **10,658** | **53.5** | **27.1** | **14.3** |
| Camargo | 12 | 22,451 | 8.3 | 1,856 | 65.7 | 19.4 | 12.8 |
| Chihuahua | 56 | 146,071 | 9.2 | 13,431 | 54.0 | 30.2 | 16.3 |
| Creel | 28 | 19,104 | 9.3 | 1,777 | 75.3 | 7.8 | 5.9 |
| Cuauhtemoc | 40 | 63,367 | 12.9 | 6,761 | 37.9 | 29.3 | 11.1 |
| Gomez Farias | 13 | 16,270 | 11.3 | 1,846 | 49.7 | 23.3 | 11.6 |
| Guachochi | 10 | 11,191 | 9.6 | 1,075 | 47.4 | 15.5 | 7.4 |
| Juarez | 36 | 124,103 | 9.6 | 11,940 | 59.8 | 26.2 | 15.7 |
| Nuevo Casas Grandes | 16 | 16,399 | 9.3 | 1,529 | 74.1 | 39.7 | 29.4 |
| Ojinaga | 4 | 9,359 | 11.0 | 1,034 | 50.2 | 9.2 | 4.6 |
| Parral | 40 | 70,334 | 9.4 | 6,608 | 48.8 | 29.6 | 14.5 |
|  |  |  |  |  |  |  |  |
| **Mexico City** | **223** | **1,348,687** | **12.2** | **18,425** | **32.4** | **40.3** | **12.8** |
| Alvaro Obregon | 17 | 101,004 | 11.8 | 11,918 | 28.0 | 50.7 | 14.2 |
| Azcapotzalco | 14 | 45,896 | 13.7 | 6,288 | 35.1 | 45.6 | 16.0 |
| Benito Juarez | 5 | 27,266 | 14.1 | 3,845 | 40.3 | 36.9 | 14.9 |
| Coyoacan | 11 | 69,887 | 11.4 | 7,967 | 30.0 | 29.9 | 9.0 |
| Cuajimalpa | 10 | 24,691 | 9.1 | 2,247 | 65.2 | 30.0 | 19.6 |
| Cuauhtemoc | 40 | 63,367 | 12.9 | 6,761 | 37.9 | 29.3 | 11.1 |
| Gustavo A. Madero | 26 | 162,568 | 13.5 | 21,947 | 32.7 | 39.7 | 13.0 |
| Iztacalco | 6 | 53,364 | 13.1 | 6,991 | 41.7 | 31.7 | 13.2 |
| Iztapalapa | 23 | 322,557 | 12.8 | 41,287 | 24.5 | 46.7 | 11.4 |
| Magdalena Contreras | 9 | 34,783 | 10.9 | 3,791 | 37.7 | 42.8 | 16.1 |
| Miguel Hidalgo | 6 | 30,255 | 13.3 | 4,024 | 74.0 | 40.9 | 30.2 |
| Milpa Alta | 10 | 42,757 | 11.5 | 4,917 | 41.5 | 38.9 | 16.1 |
| Tlahuac | 10 | 87,847 | 10.9 | 9,575 | 36.2 | 33.0 | 12.0 |
| Tlalpan | 17 | 117,849 | 10.1 | 11,903 | 28.7 | 39.7 | 11.4 |
| Venustiano Carranza | 13 | 73,769 | 12.7 | 9,369 | 24.5 | 38.2 | 9.4 |
| Xochimilco | 11 | 82,169 | 10.7 | 8,792 | 31.9 | 38.7 | 12.4 |
|  |  |  |  |  |  |  |  |
| **Coahuila de Zaragoza** | **148** | **258,925** | **12.5** | **6,059** | **10.5** | **45.9** | **4.9** |
| Acuña | 7 | 17,138 | 11.8 | 2,027 | 8.3 | 29.0 | 2.4 |
| Cuatro Cienegas | 9 | 5,046 | 11.8 | 595 | 46.6 | 30.0 | 14.0 |
| Francisco I. Madero | 26 | 36,927 | 12.1 | 4,476 | 13.3 | 64.9 | 8.6 |
| Monclova | 21 | 24,694 | 12.9 | 3,185 | 10.0 | 40.9 | 4.1 |
| Piedras Negras | 11 | 17,144 | 11.4 | 1,950 | 14.4 | 55.2 | 7.9 |
| Sabinas | 15 | 23,278 | 14.1 | 3,272 | 6.5 | 76.6 | 5.0 |
| Saltillo | 31 | 71,499 | 11.9 | 8,536 | 10.5 | 51.6 | 5.4 |
| Torreon | 30 | 59,877 | 13.4 | 8,037 | 6.8 | 21.7 | 1.5 |
|  |  |  |  |  |  |  |  |
| **Colima** | **127** | **162,196** | **11.3** | **6,726** | **39.5** | **15.7** | **6.5** |
| Colima | 53 | 73,846 | 11.6 | 8,540 | 40.8 | 16.9 | 6.9 |
| Manzanillo | 40 | 32,845 | 11.6 | 3,803 | 24.0 | 8.6 | 2.1 |
| Tecoman | 34 | 55,505 | 10.9 | 6,044 | 47.0 | 18.3 | 8.6 |
|  |  |  |  |  |  |  |  |
| **Durango** | **167** | **367,341** | **10.9** | **13,957** | **25.5** | **14.5** | **3.2** |
| Durango | 80 | 188,134 | 9.9 | 18,708 | 18.2 | 17.4 | 3.2 |
| Gomez Palacio | 51 | 94,154 | 12.2 | 11,524 | 36.6 | 7.9 | 2.9 |
| Rodeo | 18 | 24,885 | 13.1 | 3,265 | 46.3 | 4.8 | 2.2 |
| Santiago Papasquiaro | 18 | 56,404 | 10.8 | 6,095 | 23.2 | 19.0 | 4.4 |
|  |  |  |  |  |  |  |  |
| **Guanajuato** | **443** | **1,565,296** | **9.8** | **20,070** | **57.5** | **44.8** | **25.8** |
| Acambaro | 56 | 125,541 | 11.9 | 14,882 | 52.9 | 49.3 | 26.1 |
| Celaya | 71 | 252,457 | 9.8 | 24,864 | 63.0 | 39.9 | 25.2 |
| Guanajuato | 54 | 161,739 | 9.0 | 14,612 | 81.3 | 40.9 | 33.2 |
| Irapuato | 62 | 245,424 | 9.9 | 24,399 | 74.4 | 48.6 | 36.2 |
| Leon | 50 | 245,217 | 9.2 | 22,560 | 5.3 | 51.3 | 2.7 |
| Leon Delta | 39 | 152,871 | 9.5 | 14,576 | 39.4 | 0.0 | 25.4 |
| Salamanca | 49 | 210,004 | 10.5 | 21,970 | 71.6 | 42.4 | 30.4 |
| San Miguel De Allende | 60 | 172,043 | 8.6 | 14,786 | 79.7 | 40.5 | 32.3 |
|  |  |  |  |  |  |  |  |
| **Guerrero** | **951** | **1,293,050** | **11.2** | **25,393** | **46.4** | **14.0** | **6.4** |
| Acapulco | 85 | 190,446 | 12.2 | 23,234 | 34.3 | 0.0 | 3.9 |
| Centro | 257 | 271,339 | 11.4 | 28,030 | 50.5 | 12.8 | 6.5 |
| Costa Chica | 116 | 190,700 | 9.9 | 18,877 | 55.8 | 14.0 | 7.8 |
| Costa Grande | 117 | 170,938 | 11.2 | 19,223 | 45.1 | 14.4 | 6.5 |
| Montaña | 155 | 161,567 | 8.9 | 14,317 | 53.0 | 15.5 | 8.2 |
| Norte | 136 | 200,647 | 12.3 | 24,682 | 42.0 | 15.8 | 6.6 |
| Tierra Caliente | 85 | 107,413 | 12.9 | 13,825 | 41.0 | 10.5 | 4.3 |
|  |  |  |  |  |  |  |  |
| **Hidalgo** | **550** | **886,803** | **12.9** | **8,226** | **29.4** | **24.8** | **7.3** |
| Actopan | 55 | 86,523 | 13.3 | 11,507 | 37.4 | 23.5 | 8.8 |
| Apan | 22 | 34,775 | 13.3 | 4,637 | 21.3 | 18.2 | 3.9 |
| Atotonilco | 22 | 37,208 | 12.3 | 4,571 | 24.5 | 24.3 | 6.0 |
| Huejutla | 54 | 95,941 | 12.0 | 11,499 | 36.3 | 18.7 | 6.8 |
| Huichapan | 29 | 50,167 | 13.3 | 6,688 | 30.6 | 32.2 | 9.9 |
| Ixmiquilpan | 54 | 58,682 | 13.5 | 7,943 | 30.6 | 23.3 | 7.1 |
| Jacala | 19 | 28,109 | 13.0 | 3,649 | 30.6 | 15.8 | 4.8 |
| Metztitlan | 25 | 20,129 | 14.3 | 2,887 | 39.5 | 30.2 | 11.9 |
| Molango | 50 | 57,843 | 11.9 | 6,900 | 32.9 | 30.6 | 10.0 |
| Otomi Tepehua | 30 | 55,153 | 12.9 | 7,102 | 25.4 | 21.9 | 5.6 |
| Pachuca | 45 | 98,362 | 13.3 | 13,080 | 27.0 | 31.6 | 8.5 |
| Tepeji | 32 | 48,934 | 12.4 | 6,089 | 35.4 | 20.7 | 7.3 |
| Tizayuca | 19 | 46,564 | 12.1 | 5,629 | 28.5 | 21.3 | 6.1 |
| Tula | 40 | 53,769 | 13.3 | 7,154 | 24.9 | 35.1 | 8.7 |
| Tulancingo | 23 | 76,851 | 13.2 | 10,162 | 15.3 | 22.5 | 3.4 |
| Zacualtipan | 11 | 14,035 | 12.5 | 1,757 | 36.0 | 0.0 | 8.7 |
| Zimapan | 19 | 23,758 | 13.2 | 3,147 | 29.2 | 23.8 | 7.0 |
|  |  |  |  |  |  |  |  |
| **Jalisco** | **740** | **1,457,426** | **7.8** | **10,054** | **46.6** | **38.0** | **15.8** |
| Ameca | 85 | 103,472 | 7.9 | 8,191 | 60.9 | 23.2 | 14.1 |
| Autlan | 88 | 104,852 | 8.4 | 8,809 | 93.2 | 19.4 | 18.1 |
| Centro Guadalajara | 28 | 176,811 | 9.5 | 16,797 | 17.6 | 56.3 | 9.9 |
| Centro Tlaquepaque | 57 | 151,813 | 6.6 | 9,970 | 23.3 | 22.9 | 5.3 |
| Centro Tonala | 49 | 129,723 | 6.6 | 8,527 | 37.2 | 35.3 | 13.1 |
| Centro Zapopan | 50 | 191,201 | 6.5 | 12,453 | 15.3 | 57.2 | 8.7 |
| Ciudad Guzman | 67 | 100,602 | 8.5 | 8,559 | 57.8 | 30.3 | 17.5 |
| Colotlan | 62 | 34,911 | 7.3 | 2,545 | 80.0 | 33.0 | 26.4 |
| La Barca | 85 | 138,249 | 8.3 | 11,485 | 61.9 | 25.9 | 16.0 |
| Lagos De Moreno | 50 | 115,549 | 7.7 | 8,936 | 66.3 | 58.3 | 38.6 |
| Puerto Vallarta | 37 | 69,099 | 7.7 | 5,336 | 67.0 | 21.6 | 14.5 |
| Tamazula | 30 | 41,814 | 9.3 | 3,879 | 47.8 | 36.5 | 17.5 |
| Tepatitlan | 50 | 99,330 | 8.2 | 8,153 | 60.3 | 44.9 | 27.0 |
|  |  |  |  |  |  |  |  |
| **Michoacán de Ocampo** | **482** | **1,109,399** | **10.0** | **15,833** | **38.9** | **9.0** | **3.4** |
| Apatzingan | 35 | 96,895 | 10.2 | 9,923 | 29.5 | 3.2 | 0.9 |
| La Piedad | 54 | 102,792 | 11.2 | 11,514 | 40.6 | 7.3 | 3.0 |
| Lazaro Cardenas | 32 | 57,247 | 9.3 | 5,315 | 31.1 | 4.0 | 1.2 |
| Morelia | 83 | 241,572 | 9.6 | 23,249 | 35.6 | 13.5 | 4.8 |
| Patzcuaro | 66 | 131,016 | 10.0 | 13,121 | 38.5 | 7.5 | 2.9 |
| Uruapan | 50 | 135,877 | 9.6 | 13,037 | 36.1 | 14.4 | 5.2 |
| Zamora | 77 | 167,383 | 11.2 | 18,689 | 44.8 | 7.8 | 3.5 |
| Zitacuaro | 86 | 169,509 | 9.4 | 15,911 | 48.4 | 5.6 | 2.7 |
|  |  |  |  |  |  |  |  |
| **Morelos** | **206** | **538,891** | **12.1** | **24,719** | **26.8** | **16.1** | **4.4** |
| Cuautla | 82 | 215,513 | 12.0 | 25,891 | 36.0 | 16.9 | 6.1 |
| Cuernavaca | 81 | 237,352 | 12.1 | 28,714 | 22.4 | 17.6 | 3.9 |
| Jojutla | 43 | 86,026 | 12.5 | 10,763 | 16.3 | 10.0 | 1.6 |
|  |  |  |  |  |  |  |  |
| **Mexico State** | **1,078** | **3,409,474** | **9.4** | **20,212** | **37.4** | **17.4** | **6.2** |
| Amecameca | 69 | 338,708 | 8.9 | 30,035 | 29.9 | 23.8 | 7.1 |
| Atizapan De Zaragoza | 27 | 124,329 | 8.5 | 10,552 | 25.7 | 31.7 | 8.1 |
| Atlacomulco | 74 | 101,789 | 9.2 | 9,357 | 56.6 | 15.2 | 8.6 |
| Cuautitlan | 33 | 197,765 | 8.9 | 17,523 | 17.6 | 26.3 | 4.6 |
| Ecatepec | 25 | 269,465 | 10.7 | 28,833 | 36.8 | 9.9 | 3.6 |
| Ixtlahuaca | 148 | 230,806 | 8.8 | 20,389 | 37.4 | 15.9 | 6.0 |
| Jilotepec | 77 | 110,482 | 9.3 | 10,233 | 73.0 | 22.6 | 16.5 |
| Naucalpan | 20 | 116,371 | 9.7 | 11,322 | 24.6 | 33.4 | 8.2 |
| Nezahualcoyotl | 23 | 254,077 | 11.0 | 27,848 | 32.4 | 23.5 | 7.6 |
| Tejupilco | 71 | 96,804 | 9.5 | 9,163 | 42.7 | 24.4 | 10.4 |
| Tenancingo | 82 | 169,966 | 9.3 | 15,860 | 53.8 | 12.0 | 6.5 |
| Tenango Del Valle | 46 | 116,984 | 9.5 | 11,079 | 39.4 | 14.9 | 5.9 |
| Teotihuacan | 46 | 107,613 | 9.1 | 9,759 | 58.1 | 18.3 | 10.6 |
| Texcoco | 63 | 307,250 | 9.6 | 29,582 | 39.3 | 10.2 | 4.0 |
| Tlalnepantla | 19 | 91,551 | 10.2 | 9,338 | 36.1 | 22.5 | 8.1 |
| Toluca | 78 | 299,095 | 9.3 | 27,917 | 13.7 | 11.0 | 1.5 |
| Valle De Bravo | 60 | 132,320 | 8.5 | 11,289 | 37.2 | 6.0 | 2.2 |
| Xonacatlan | 71 | 196,968 | 9.0 | 17,681 | 42.8 | 14.2 | 6.1 |
| Zumpango | 51 | 142,363 | 8.3 | 11,870 | 72.1 | 10.0 | 7.2 |
|  |  |  |  |  |  |  |  |
| **Nayarit** | **208** | **285,409** | **10.7** | **15,386** | **22.5** | **46.9** | **10.5** |
| Compostela | 39 | 82,818 | 10.3 | 8,531 | 23.2 | 44.6 | 10.3 |
| Tepic | 53 | 103,524 | 9.7 | 9,997 | 23.2 | 42.4 | 9.8 |
| Tuxpan | 116 | 99,067 | 12.2 | 12,092 | 21.2 | 53.4 | 11.3 |
|  |  |  |  |  |  |  |  |
| **Nuevo León** | **431** | **513,227** | **13.2** | **10,526** | **28.3** | **43.8** | **12.3** |
| Cadereyta Jimenez | 42 | 37,196 | 12.7 | 4,741 | 33.6 | 36.8 | 12.4 |
| Doctor Arroyo | 95 | 54,130 | 13.6 | 7,353 | 45.6 | 38.9 | 17.7 |
| Guadalupe | 63 | 112,201 | 12.2 | 13,672 | 18.6 | 34.9 | 6.5 |
| Montemorelos | 71 | 51,082 | 14.3 | 7,296 | 35.7 | 43.7 | 15.6 |
| Monterrey Norte | 44 | 81,613 | 11.9 | 9,671 | 22.2 | 61.7 | 13.7 |
| Sabinas Hidalgo | 41 | 25,386 | 13.1 | 3,325 | 35.5 | 52.1 | 18.5 |
| San Pedro Garza Garcia | 41 | 44,704 | 10.4 | 4,634 | 33.9 | 46.2 | 15.6 |
|  |  |  |  |  |  |  |  |
| **Oaxaca** | **802** | **1,307,705** | **10.2** | **24,093** | **35.5** | **6.1** | **1.6** |
| Costa | 96 | 189,673 | 9.2 | 17,503 | 34.9 | 3.2 | 1.1 |
| Istmo | 127 | 205,880 | 11.7 | 24,172 | 36.8 | 2.5 | 0.9 |
| Mixteca | 160 | 156,099 | 9.8 | 15,374 | 34.0 | 3.7 | 1.3 |
| Sierra | 108 | 97,769 | 10.3 | 10,115 | 70.5 | 1.7 | 1.2 |
| Tuxtepec | 69 | 175,660 | 11.3 | 19,774 | 26.4 | 2.3 | 0.6 |
| Valles Centrales | 242 | 376,215 | 9.6 | 36,140 | 38.2 | 6.0 | 2.3 |
|  |  |  |  |  |  |  |  |
| **Puebla** | **673** | **1,625,491** | **9.1** | **17,355** | **36.0** | **43.9** | **14.9** |
| Acatlan | 22 | 42,029 | 10.2 | 4,282 | 27.7 | 40.9 | 11.3 |
| Chignahuapan | 50 | 88,780 | 7.8 | 6,898 | 56.7 | 46.5 | 26.3 |
| Huauchinango | 60 | 144,962 | 9.3 | 13,475 | 48.3 | 27.2 | 13.1 |
| Huejotzingo | 95 | 255,118 | 9.3 | 23,635 | 42.3 | 27.7 | 11.7 |
| Izucar De Matamoros | 45 | 77,091 | 10.2 | 7,829 | 37.7 | 34.7 | 13.1 |
| Puebla | 42 | 238,031 | 8.7 | 20,709 | 31.3 | 48.4 | 15.2 |
| San Salvador El Seco | 79 | 143,693 | 9.5 | 13,617 | 29.9 | 82.1 | 24.6 |
| Tehuacan | 68 | 207,306 | 9.0 | 18,754 | 21.2 | 34.0 | 7.2 |
| Tepexi De Rodriguez | 120 | 230,995 | 8.8 | 20,270 | 27.2 | 68.3 | 18.6 |
| Zacapoaxtla | 91 | 177,711 | 9.1 | 16,116 | 50.4 | 28.8 | 14.6 |
|  |  |  |  |  |  |  |  |
| **Querétaro** | **252** | **407,080** | **7.7** | **9,450** | **71.9** | **18.3** | **13.1** |
| Cadereyta De Montes | 64 | 73,115 | 8.0 | 5,837 | 94.2 | 15.6 | 14.7 |
| Jalpan De Serra | 45 | 41,833 | 8.1 | 3,392 | 80.9 | 35.0 | 28.3 |
| Queretaro | 70 | 169,491 | 7.1 | 12,045 | 59.8 | 19.3 | 11.5 |
| San Juan Del Rio | 74 | 122,641 | 8.2 | 10,084 | 72.4 | 12.7 | 9.2 |
|  |  |  |  |  |  |  |  |
| **Quintana Roo** | **185** | **277,187** | **8.6** | **9,210** | **79.2** | **18.3** | **15.6** |
| Cancun | 43 | 150,813 | 7.8 | 11,819 | 81.1 | 11.6 | 9.4 |
| Chetumal | 82 | 74,379 | 9.1 | 6,749 | 81.1 | 22.8 | 23.5 |
| Felipe Carrillo Puerto | 59 | 51,995 | 9.9 | 5,166 | 71.0 | 31.2 | 22.1 |
|  |  |  |  |  |  |  |  |
| **San Luis Potosí** | **291** | **714,735** | **10.9** | **12,078** | **45.5** | **28.0** | **12.6** |
| Ciudad Valles | 41 | 83,363 | 12.7 | 10,570 | 49.8 | 26.4 | 13.2 |
| Matehuala | 42 | 64,395 | 10.6 | 6,835 | 35.3 | 32.8 | 11.6 |
| Rioverde | 46 | 107,894 | 12.1 | 13,012 | 46.7 | 28.3 | 13.2 |
| San Luis Potosi | 45 | 155,478 | 10.6 | 16,502 | 46.6 | 27.2 | 12.7 |
| Soledad De Graciano Sanchez | 42 | 107,456 | 10.0 | 10,695 | 44.3 | 31.6 | 14.0 |
| Tamazunchale | 42 | 102,537 | 10.8 | 11,028 | 49.5 | 28.0 | 13.9 |
| Tancanhuitz | 33 | 74,243 | 10.2 | 7,557 | 51.0 | 20.3 | 10.3 |
|  |  |  |  |  |  |  |  |
| **Sinaloa** | **266** | **531,099** | **10.8** | **10,871** | **27.6** | **26.0** | **6.8** |
| Culiacan | 74 | 145,122 | 10.3 | 14,975 | 22.7 | 41.7 | 9.5 |
| Escuinapa | 15 | 41,114 | 10.4 | 4,288 | 25.4 | 24.4 | 6.2 |
| Guamuchil | 40 | 52,937 | 11.9 | 6,283 | 19.2 | 29.9 | 5.7 |
| Guasave | 49 | 104,026 | 11.0 | 11,475 | 33.7 | 13.2 | 4.4 |
| Los Mochis | 43 | 96,041 | 10.2 | 9,798 | 28.6 | 12.8 | 3.7 |
| Mazatlan | 46 | 91,859 | 11.3 | 10,414 | 33.2 | 28.1 | 9.3 |
|  |  |  |  |  |  |  |  |
| **Sonora** | **207** | **420,723** | **12.2** | **10,386** | **25.6** | **24.7** | **6.6** |
| Caborca | 13 | 22,125 | 11.8 | 2,603 | 34.0 | 14.1 | 4.8 |
| Ciudad Obregon | 33 | 90,518 | 12.9 | 11,689 | 18.2 | 30.3 | 5.5 |
| Hermosillo | 62 | 116,952 | 11.3 | 13,257 | 38.1 | 29.4 | 11.2 |
| Navojoa | 61 | 89,825 | 13.2 | 11,892 | 18.2 | 18.5 | 3.4 |
| San Luis Rio Colorado | 4 | 42,460 | 12.7 | 5,386 | 30.6 | 31.6 | 9.7 |
| Santa Ana | 33 | 57,356 | 11.2 | 6,417 | 16.7 | 14.9 | 2.5 |
|  |  |  |  |  |  |  |  |
| **Tabasco** | **584** | **766,993** | **11.5** | **13,514** | **47.1** | **26.3** | **11.7** |
| Balancan | 20 | 28,513 | 11.9 | 3,393 | 52.0 | 18.4 | 9.6 |
| Cardenas | 34 | 75,641 | 12.3 | 9,304 | 31.2 | 19.2 | 6.0 |
| Centla | 21 | 44,489 | 10.6 | 4,716 | 50.6 | 20.9 | 10.6 |
| Centro | 257 | 271,339 | 11.4 | 28,030 | 50.5 | 12.8 | 6.5 |
| Comalcalco | 31 | 71,151 | 11.6 | 8,254 | 76.2 | 16.4 | 12.5 |
| Cunduacan | 28 | 50,907 | 11.5 | 5,854 | 48.0 | 20.5 | 9.8 |
| Emiliano Zapata | 7 | 13,685 | 13.0 | 1,779 | 37.1 | 27.4 | 10.2 |
| Huimanguillo | 37 | 65,675 | 10.6 | 6,962 | 42.7 | 52.1 | 22.3 |
| Jalapa | 13 | 16,827 | 12.6 | 2,120 | 56.9 | 8.9 | 5.0 |
| Jalpa De Mendez | 22 | 29,477 | 10.0 | 2,948 | 33.0 | 79.0 | 26.1 |
| Jonuta | 13 | 16,940 | 12.2 | 2,067 | 51.5 | 15.1 | 7.8 |
| Macuspana | 45 | 71,231 | 13.0 | 9,260 | 33.4 | 18.1 | 6.1 |
| Nacajuca | 23 | 38,258 | 9.2 | 3,520 | 44.9 | 55.2 | 24.8 |
| Paraiso | 11 | 30,098 | 11.0 | 3,311 | 48.4 | 12.7 | 6.2 |
| Tacotalpa | 20 | 23,436 | 10.8 | 2,531 | 40.5 | 51.4 | 20.8 |
| Teapa | 15 | 24,675 | 12.5 | 3,084 | 40.6 | 37.9 | 15.4 |
| Tenosique | 17 | 22,807 | 11.8 | 2,691 | 57.1 | 31.8 | 18.1 |
|  |  |  |  |  |  |  |  |
| **Tamaulipas** | **296** | **641,955** | **14.0** | **8,990** | **34.8** | **32.3** | **10.7** |
| Altamira | 24 | 67,368 | 12.9 | 8,718 | 30.2 | 76.8 | 23.2 |
| Jaumave | 18 | 23,613 | 14.1 | 3,319 | 26.4 | 19.4 | 5.1 |
| Mante | 26 | 51,780 | 16.0 | 8,287 | 33.7 | 26.2 | 8.8 |
| Matamoros | 45 | 82,712 | 13.8 | 11,414 | 36.4 | 26.6 | 9.7 |
| Miguel Aleman | 14 | 20,460 | 13.9 | 2,841 | 28.2 | 51.6 | 14.5 |
| Nuevo Laredo | 16 | 43,724 | 12.8 | 5,597 | 47.3 | 17.1 | 8.1 |
| Padilla | 21 | 31,674 | 14.3 | 4,518 | 36.9 | 40.4 | 14.9 |
| Reynosa | 28 | 80,434 | 13.7 | 11,019 | 45.2 | 16.4 | 7.4 |
| San Fernando | 22 | 31,100 | 14.1 | 4,399 | 34.0 | 49.5 | 16.8 |
| Tampico | 20 | 69,048 | 14.9 | 10,316 | 34.2 | 19.9 | 6.8 |
| Valle Hermoso | 25 | 45,000 | 13.9 | 6,244 | 30.3 | 13.5 | 4.1 |
| Victoria | 37 | 95,042 | 14.1 | 13,363 | 28.5 | 38.0 | 10.8 |
|  |  |  |  |  |  |  |  |
| **Tlaxcala** | **123** | **433,665** | **9.2** | **15,412** | **62.5** | **17.7** | **9.8** |
| Apizaco | 46 | 130,472 | 8.7 | 11,357 | 70.3 | 0.0 | 8.3 |
| Huamantla | 24 | 84,302 | 9.0 | 7,599 | 62.7 | 19.4 | 12.1 |
| Tlaxcala | 53 | 218,891 | 9.5 | 20,838 | 57.9 | 17.0 | 9.8 |
|  |  |  |  |  |  |  |  |
| **Veracruz** | **793** | **2,038,825** | **11.6** | **23,174** | **26.0** | **34.6** | **8.8** |
| Coatzacoalcos | 76 | 253,375 | 11.6 | 29,281 | 26.0 | 33.6 | 8.7 |
| Cordoba | 67 | 201,865 | 10.9 | 22,061 | 30.9 | 34.5 | 10.6 |
| Cosamaloapan | 44 | 128,660 | 12.8 | 16,495 | 20.8 | 67.9 | 14.2 |
| Martinez De La Torre | 52 | 154,669 | 12.0 | 18,604 | 28.3 | 29.1 | 8.2 |
| Orizaba | 63 | 168,053 | 10.3 | 17,287 | 30.6 | 32.1 | 9.9 |
| Panuco | 63 | 142,338 | 10.8 | 15,434 | 25.6 | 24.2 | 6.2 |
| Poza Rica | 92 | 211,180 | 11.5 | 24,359 | 25.3 | 26.4 | 6.7 |
| San Andres Tuxtla | 54 | 198,086 | 12.8 | 25,385 | 25.0 | 33.5 | 8.4 |
| Tuxpan | 116 | 99,067 | 12.2 | 12,092 | 21.2 | 53.4 | 11.3 |
| Veracruz | 60 | 180,043 | 12.5 | 22,580 | 23.7 | 28.4 | 6.7 |
| Xalapa | 83 | 266,111 | 10.5 | 28,005 | 26.4 | 32.5 | 8.6 |
|  |  |  |  |  |  |  |  |
| **Yucatán** | **166** | **451,156** | **10.4** | **16,984** | **39.8** | **23.8** | **9.0** |
| Merida | 66 | 192,025 | 11.0 | 21,130 | 37.1 | 26.5 | 9.8 |
| Ticul | 32 | 78,011 | 10.1 | 7,845 | 46.4 | 18.6 | 8.6 |
| Valladolid | 68 | 137,610 | 9.9 | 13,572 | 49.4 | 20.1 | 9.9 |
|  |  |  |  |  |  |  |  |
| **Zacatecas** | **234** | **469,740** | **10.9** | **8,775** | **40.0** | **14.7** | **5.6** |
| Concepcion Del Oro | 21 | 26,242 | 11.7 | 3,070 | 41.0 | 6.3 | 2.6 |
| Fresnillo | 47 | 108,317 | 10.9 | 11,795 | 42.5 | 9.1 | 3.9 |
| Jalpa | 24 | 47,881 | 12.1 | 5,786 | 55.1 | 14.4 | 7.9 |
| Ojocaliente | 53 | 104,896 | 10.1 | 10,579 | 41.1 | 11.8 | 4.9 |
| Rio Grande | 29 | 49,424 | 11.7 | 5,806 | 36.6 | 18.5 | 6.8 |
| Tlaltenango | 25 | 46,844 | 12.5 | 5,844 | 38.0 | 24.7 | 9.4 |
| Zacatecas | 36 | 75,895 | 9.6 | 7,264 | 32.4 | 18.9 | 6.1 |

MOH, Ministry of Health; PHC, primary health center; T2D, type 2 diabetes.
